# The impact of high grade glial neoplasms on human cortical electrophysiology

**DOI:** 10.1371/journal.pone.0173448

**Published:** 2017-03-20

**Authors:** S. Kathleen Bandt, Jarod L. Roland, Mrinal Pahwa, Carl D. Hacker, David T. Bundy, Jonathan D. Breshears, Mohit Sharma, Joshua S. Shimony, Eric C. Leuthardt

**Affiliations:** 1 Department of Neurological Surgery, Yale University School of Medicine, New Haven, Connecticut, United States of America; 2 Department of Neurological Surgery, Washington University, St. Louis, Missouri, United States of America; 3 Department of Biomedical Engineering, Washington University, St. Louis, Missouri, United States of America; 4 Washington University School of Medicine, St. Louis, Missouri, United States of America; 5 Department of Rehabilitation Medicine, University of Kansas, Kansas City, Kansas, United States of America; 6 Department of Neurological Surgery, University of California San Francisco, San Francisco, California, United States of America; 7 Mallinckrodt Institute of Radiology, Washington University School of Medicine, St. Louis, Missouri, United States of America; 8 Center for Innovation in Neuroscience and Technology, Washington University School of Medicine, St. Louis, Missouri, United States of America; 9 Brain Laser Center, Washington University School of Medicine, St. Louis, Missouri, United States of America; University of California Los Angeles, UNITED STATES

## Abstract

**Objective:**

The brain’s functional architecture of interconnected network-related oscillatory patterns in discrete cortical regions has been well established with functional magnetic resonance imaging (fMRI) studies or direct cortical electrophysiology from electrodes placed on the surface of the brain, or electrocorticography (ECoG). These resting state networks exhibit a robust functional architecture that persists through all stages of sleep and under anesthesia. While the stability of these networks provides a fundamental understanding of the organization of the brain, understanding how these regions can be perturbed is also critical in defining the brain’s ability to adapt while learning and recovering from injury.

**Methods:**

Patients undergoing an awake craniotomy for resection of a tumor were studied as a unique model of an evolving injury to help define how the cortical physiology and the associated networks were altered by the presence of an invasive brain tumor.

**Results:**

This study demonstrates that there is a distinct pattern of alteration of cortical physiology in the setting of a malignant glioma. These changes lead to a physiologic sequestration and progressive synaptic homogeneity suggesting that a de-learning phenomenon occurs within the tumoral tissue compared to its surroundings.

**Significance:**

These findings provide insight into how the brain accommodates a region of “defunctionalized” cortex. Additionally, these findings may have important implications for emerging techniques in brain mapping using endogenous cortical physiology.

## Introduction

The brain’s functional architecture of interconnected network-related oscillatory patterns in discrete cortical regions has been well established [1, 2]. The signal modalities identifying these networked regions have largely been either resting-state metabolic activity studied with functional magnetic resonance imaging (fMRI) studies [1–8] or direct cortical electrophysiology from electrodes placed on the surface of the brain, or electrocorticography (ECoG) [2, 9–12]. One of the earliest and most robust links between cortical electrophysiological signals and the radiographic blood oxygenation level dependent (BOLD) signal are those physiologic signals within the infraslow frequency band (<0.5 Hz) or the slow cortical potential (SCP) [2]. These resting state SCP networks exhibit a robust functional architecture that persists through all stages of sleep and under anesthesia and represent the physiologic correlate of the BOLD imaging signal [2, 13]. While the stability of these networks provides a fundamental understanding of the organization of the brain, understanding how these regions can be perturbed or altered is also critical in defining the brain’s ability to adapt while learning and recovering from injury.

Deliberate training and learning appears to strengthen resting state connectivity between brain regions that are mutually engaged in a learning task [14]. FMRI studies in animal models of stroke and in human patients recovering from cerebrovascular insults also have shown evidence to suggest that rehabilitation can alter resting state connectivity after injury [15–17]. These findings highlight that these networks, while quite durable through multiple states of consciousness, are not immutable. Namely, that with intensive stimuli they can be subject to augmentation (in the case of learning) or restoration (in the context of injury). However, how the brain responds to a defunctionalized region is incompletely understood. The manner in which resting state functional architecture is altered in the context of an invasive brain tumor has been explored in a small number of studies using magnetoencephalography[18, 19] and more recently using resting state functional MRI[20], however this question has never been investigated using invasively acquired ECoG. The limited experience investigating the impact of an invasive glioma on resting state networks is referable in part to technical barriers with functional imaging in the context of an invasive lesion, specifically the decoupling effect these lesions exert on the neurovascular relationship which can make interpretation of their imaging more challenging [20–23]. While invasive cortical physiology has been used to identify and study resting state networks associated with functional networks such as motor and language [9, 24, 25], this modality has not been used in the context of a pathologic lesion.

To address this gap, patients undergoing awake craniotomies for resection of a tumor were studied as a unique model to define how the cortical physiology and the associated networks were altered by the presence of an invasive brain tumor. Distinct from acute injuries such as a stroke, which immediately and radically reduce the function of a region of brain, tumors that invade the brain can provide insights into how the cortex accommodates to a subregion becoming non-functional. In this subacute injury model, we hypothesized that in the setting of an infiltrative glioblastoma, resting state connectivity would be perturbed in the cortex. This study demonstrates that there is a distinct pattern of alteration of cortical physiology in the setting of a malignant glioma. These changes lead to a physiologic sequestration and progressive synaptic homogeneity suggesting that a de-learning phenomenon occurs within the tumoral tissue compared to its surroundings.

## Materials and methods

### Subjects

**Fig 1:**
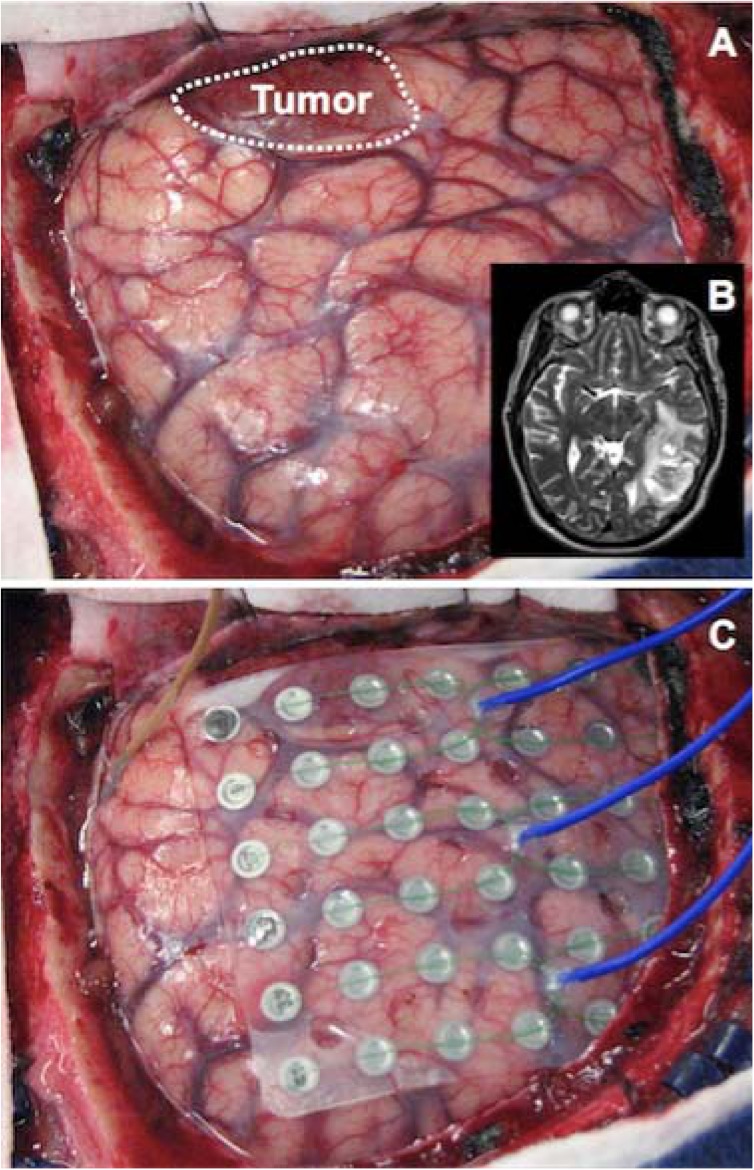
Intraoperative Photograph. (A) Exposed cortical surface demonstrating gross tumor. (B) Axial MRI slice from same patient identifying left temporo-occipital tumor. (C) Intraoperative ECoG grid on cortical surface for mapping.

**Fig 2:**
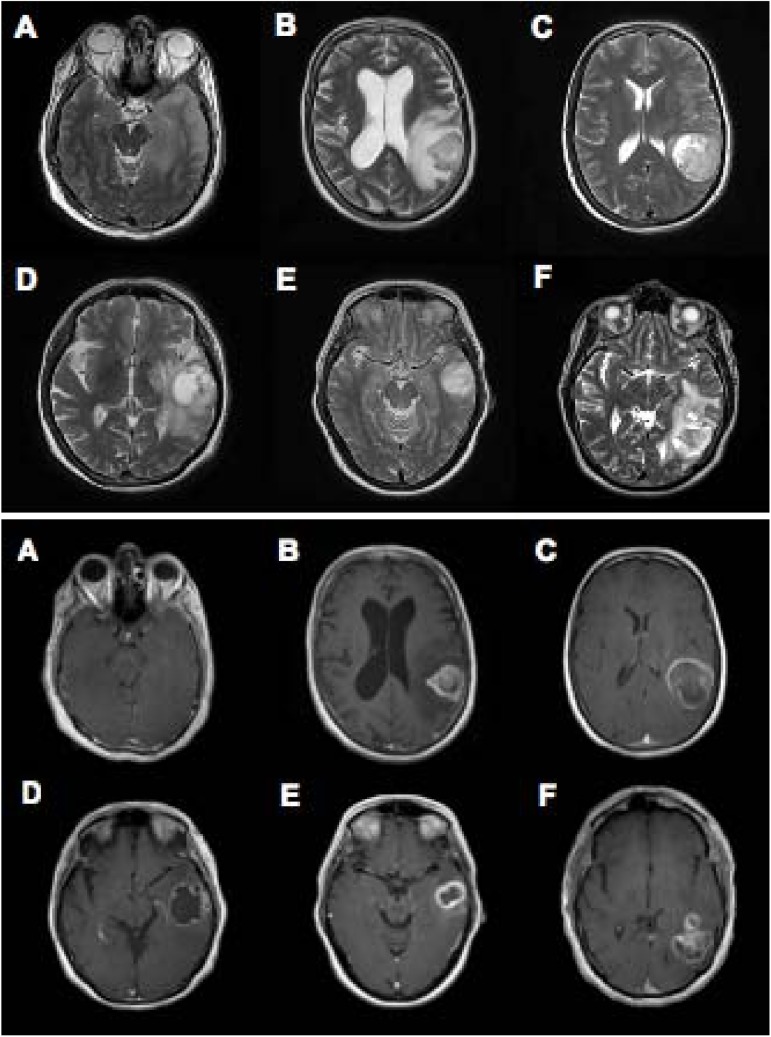
Preoperative MR Imaging. Representative axial slices demonstrating preoperative T2 weighted (upper panel) and contrast enhanced T1 weighted (lower panel) MR imaging demonstrating intracranial neoplasm in each patient.

Six patients undergoing surgical treatment for glioblastoma were included in this study. Study protocol was approved by the Human Research and Protection Organization at Washington University School of Medicine. Before inclusion, all patients provided written informed consent for study participation. Exclusion criteria included the absence of a glioblastoma on pathologic analysis of neoplastic tissue. See Table 1 for demographic and clinical information. Each patient underwent craniotomy for the resection of their tumor. See Figs 1 and 2 for intraoperative photo and preoperative imaging respectively. Following acquisition of a baseline ECoG signal, surgical resection of the patient’s neoplastic tissue proceeded in the usual fashion.

**Table 1:** Patient Demographics & Clinical Information.

Patient	Age	Gender	Handedness	Pathology	Location	Preoperative Neuro Exam	Postoperative Neuro Exam
A	48	M	Right	GBM	L Temporal	Confused, expressive dysphasia	Improved; fluent speech with subtle naming deficit
B	64	F	Right	GBM	L Parietal	Confused, dysgraphia	Improved; normal exam
C	54	F	Right	GBM	L Parietal	Expressive dysphasia, dyscalculia, dysgraphia, R/L confusion	Improved; persistent subtle expressive dysphasia
D	77	F	Right	GBM	L Temporal	Expressive dysphasia	Improved, intermittent paraphasias
E	69	F	Right	GBM	L Temporal	History of acute 6wks prior to presentation, resolved at time of presentation	Normal exam
F	55	M	Right	GBM	L Temporo-occipital	Expressive dysphasia	Stable; expressive dysphasia

GBM = Glioblastoma; M = Male; F = Female; R = Right; L = Left.

### Data collection

With the patient sedated with remifentanil and midazolam for surgical exposure prior to planned awake craniotomy, the cortical surface electrode array was connected to amplifiers and an additional 1x4 cortical electrode strip was placed to provide the reference and ground. Five minutes of baseline cortical signal was acquired with the patient sedated prior to wake-up for planned awake tumor resection. Electrode grid placement was selected based on the anatomical relationship between the tumor and local functional cortex.

ECoG signals were acquired from the electrode array (Ad-Tech Medical Instrument Corporation, Racine WI, USA) at a sampling frequency of 1,200 Hz using g.tech biosignal amplifiers (Guger Technologies, Graz, Austria). The data were stored on a Dell PC running BCI2000 software [26]. The ECoG electrode array contained either 48 or 64 2.3mm diameter platinum electrodes spaced 10mm apart. Patient data is available in Supporting Information, S1–S12 Files.

### Electrode selection

Electrodes were defined as overlying tumor, peritumor or distant cortex based on analysis of both intraoperative photography and the patient’s preoperative MRI. In 4 patients, grossly neoplastic tissue presented to the cortical surface. In these cases, intraoperative photography was used exclusively to define electrodes overlying tumoral tissue. Peritumoral tissue was defined as cortex underlying perimeter electrodes immediately adjacent to tumor electrodes located within 1 cm of tumoral tissue. Distant electrodes were defined as all remaining electrodes. In the case of the 2 patients whose tumor did not present to the cortical surface, intraoperative photography was used to define the orientation of the grid relative to the exposed cortical surface. This was then related to the patient’s preoperative MRI to define the margins of the tumor relative to the exposed cortex. Electrodes were again defined as overlying tumoral tissue, peritumoral cortex or distant cortex in the same way. Electrodes determined to be overlying peritumor cortex were excluded from further analysis due to their position as a relative transition zone between the tumor and distant cortex.

### Signal analysis

Signals from every electrode were visually inspected and those electrodes identified as having poor signal to noise characteristics (amplitude greater than 10x that of the majority of electrodes in the grid) were excluded from further analysis (33 of 304 electrodes, 11%). Remaining signals were re-referenced to the amplifier-specific common mean for further analysis. The 5-minute acquisition was analyzed as a single time epoch.

Power analysis was performed by Welch’s Method [27]. The time-varying power was computed for frequencies ranging from 0.5 to 150 Hz. Known noise bands at 60 and 120 Hz were avoided by band-pass filtering 2 Hz bands centered on each known noise peak. Analysis was cut off at 150 Hz as determined by the signal’s noise floor. Analysis of trends in the absolute band power and the variance in absolute band power followed for the subdivided “tumor” and “distant” signals. Findings between these two source signals were then compared. Power spectral curves were then fit to a power-law function by converting frequency and power to logarithmic space and fitting a linear regression curve. ECoG data characteristically has a knee in the low-frequency alpha/beta range and therefore the power-law structure was fit after this observed knee [28]. The error was determined by summation of squares of difference between the fit and observed curves. The ratio of these sum-of-square errors with observed variance was calculated to produce R^2^ values, which represent the percentage of variability explained by the power-law spectral model regression fit (Table 2). The R^2^ values quantify a goodness-of-fit supporting maintenance of the power-law structure in both tumor and distant electrodes.

**Table 2:** Power-law spectral fit.

Patient	Tumor R^2^ values	Distant R^2^ value
A	0.989	0.982
B	0.732	0.914
C	0.976	0.991
D	0.991	0.988
E	0.991	0.982
F	0.907	0.968

Networked interactions were also evaluated. Signals from every electrode were first visually inspected and those electrodes identified as noisy (as above) were excluded from further analysis. Signals from the remaining electrodes were re-referenced to the global mean and low-pass filtered using a 3rd order lowpass digital Butterworth filter (cutoff = 0.5Hz) to isolate the SCP. The temporal correlation of the SCP over the five-minute epoch was then calculated for all electrode pairs by first computing the covariance (Eq 1) between each pair.

$$\mathrm{Covariance}(m,n)=\sum_{i}^{N}\frac{{(\mathrm{electrode}}_{i}^{m}-\bar{{\mathrm{electrode}}^{m}}){(\mathrm{electrode}}_{i}^{n}-\bar{{\mathrm{electrode}}^{n}})}{N}$$ (1)

Here, *electrode*^*m*^_*i*_ and *electrode*^*n*^_*i*_ represent the *i*^th^ of *N* values of the SCP signal from the *m*^*th*^ and *n*^*th*^ electrodes, respectively. The correlation coefficient between each electrode pair was then obtained by Eq (2).

$$Correlation\;Coefficient(m,n)=\frac{Covariance(m,n)}{\sqrt{Covariance(m,m)Covariance(n,n)}}$$ (2)

Electrical potentials measured from the surface of the brain have a distance-dependent inherent correlation strength[29, 30]. These local spatial correlations tend to be stronger in lower frequencies than higher frequencies[31]. Here, we investigate low frequency correlations within relatively confined regions defined by the presence or absence of a tumor. To control for this effect of local spatial correlation, we employed a method of distance regression of the correlation values between electrode pairs. Prior literature suggests the relationship is not entirely linear[31], therefore, we fit a spline regression curve to the distance and correlation coefficients of electrode pairs. The fitted regression was then subtracted from each correlation value and this corrected value, detrended for distance, was used for further analysis.

Next, ECoG signal was subdivided into those signals recorded from “tumor” electrodes and signals recorded from “distant” electrodes. For each electrode grouping, tumor and distant, a central, non-noisy electrode channel was selected as a representative seed electrode. First, we investigated mean ECoG correlation strength among tumor electrodes compared to distant electrodes to evaluate for alteration of signaling capacity within tumor-infiltrated brain. In this comparison, positive and negative correlations were considered to have equal meaning with respect to the strength of shared information, therefore, the absolute value of the correlation coefficient was used. The correlation between the seed electrode and all other electrodes within each group was calculated for each patient. These correlation values were then averaged across all electrode pairs within each group and compared using a two-sample t-test for each patient. The Bonferroni correction for number of patients was applied to correct for multiple comparisons.

Next, we tested the distribution of correlations for each region. Pairs of tumor electrodes and distant electrodes were evaluated as a group to determine if they represented samples of significantly different distributions. The Kolmogorov-Smirnov test of difference in sample distributions was used for this purpose. To correct for fewer electrodes overlying tumor compared to distant cortical regions in each subject, we employed a permutation resampling method to achieve equal sample size within each group. Correlations where binned by distance between electrode pairs for each group. Observed samples where then resampled for each group to achieve equal numbers of channel pairs with respect to binned distance. The distribution of correlations was normalized for each group and reported by frequency count area-under-the-curve (AUC).

Finally, topographical correlation maps relative to the seed electrode were computed for each patient and each electrode group. The Pearson correlation coefficient of each electrode with the seed electrode was computed and plotted in a grid configuration matching that of the ECoG electrode array. This was repeated for all pairs of electrodes with the tumor seed and distant seed, respectively. The resulting correlation maps provide a measure of topography of connectivity within the tumor and distant regions. To quantify the relationship between tumor and distant topographies, we calculated spatial correlation coefficients between tumor and distant topography maps for each patient. The spatial correlation is performed similar to the temporal correlation described in Eq 1, iterating over electrodes at successive locations instead of ECoG samples at successive time points. Positive correlations represent strength of similarity in topography, while negative correlations represent strength of reciprocal topography, such as elevations in one map corresponding to depression in the other.

## Results

### Alterations in power spectra

**Fig 3:**
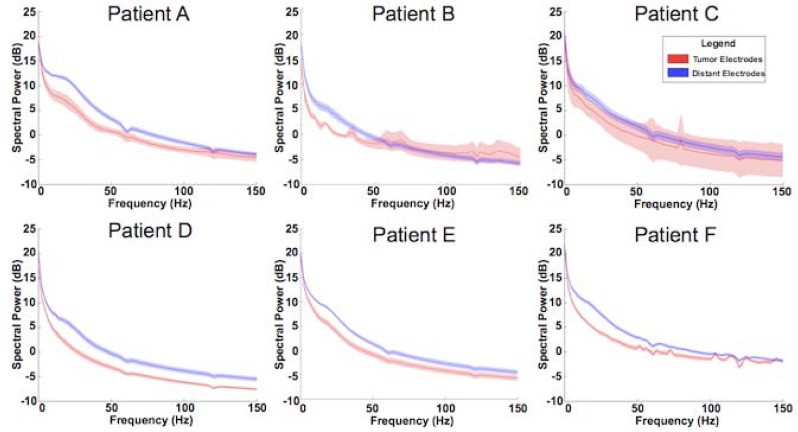
Power spectra comparing tumor (red) and distant (blue) spectral densities from each individual subject.

Global power was analyzed from 0.5–150 Hz. A consistent reduction in overall power was identified for tumor electrodes when compared against distant electrodes. Statistical significance across subjects as indicated by 95% confidence intervals was reached in 5 of 6 subjects (Fig 3). The remaining subject’s power spectra appeared consistent with those reaching statistical significance but noise inherent within the intraoperative setting limited the ability to achieve statistical significance. This power reduction was seen across all frequency bands suggesting maintenance of power-law scaling in these signals despite an overall reduction in amplitude. Thus, while tumors lead to a decrease in global cortical activity, they do not result in a fundamental change in the nature of the scaling relationship.

### Maintenance of connectivity

**Fig 4:**
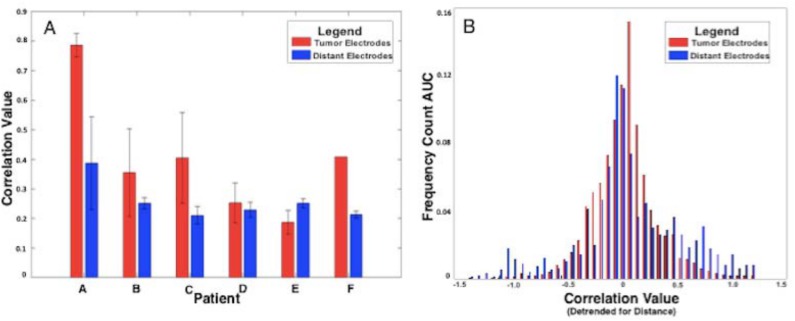
A. Bar plot demonstrating comparison between average correlation values between all tumor electrode pairs (red) and all distant electrode pairs (blue). This suggests that connectivity is maintained within cortical regions invaded by glioma. B. Bar histogram demonstrating comparison between distributions of correlation values (detrended for distance) between tumor electrodes (red) and a multiply permuted and resampled subpopulation of distant electrodes (blue) normalized to their respective probability density functions. This accounts for differences in the number of tumor electrodes compared to distant electrodes as well as smaller inter-electrode differences in the tumor electrode subgroup. There was no significant difference between the two groups

Cortical connectivity, defined by correlation of SCP oscillations, was maintained when comparing time series data from electrodes overlying neoplastic versus distant cortical tissue. Correlation strength among tumor electrodes did not demonstrate a decrease among pairs of tumor electrodes relative to distant electrodes as would be expected if SCP connectivity was disrupted by the tumor (Fig 4A). Likewise, comparison between the distributions of correlation values across electrode pairs for both tumor and distant regions did not demonstrate a statistically significant difference as determined by a two-sample Kolmogorov-Smirnov test, p>0.05. This supports the notion that SCP connectivity is not reduced by the presence of a tumor (Fig 4B). It should be noted that the effect of regressing distance from correlations may artificially elevate correlation coefficients above 1.0 or below -1.0 due to intrinsically strong correlations despite a large distance. This corrected correlation value maintains the same properties of a standard correlation coefficient, namely being a dimensionless quantity that provides a relative measure of correlation strength and can be interpreted as such despite slight deviations from the standard, expected range of correlation values. The tumor and distant group correlation distributions are presented in the corrected form as correlation values which have been detrended for distance (Fig 4B).

### Disruption of local topography

**Fig 5:**
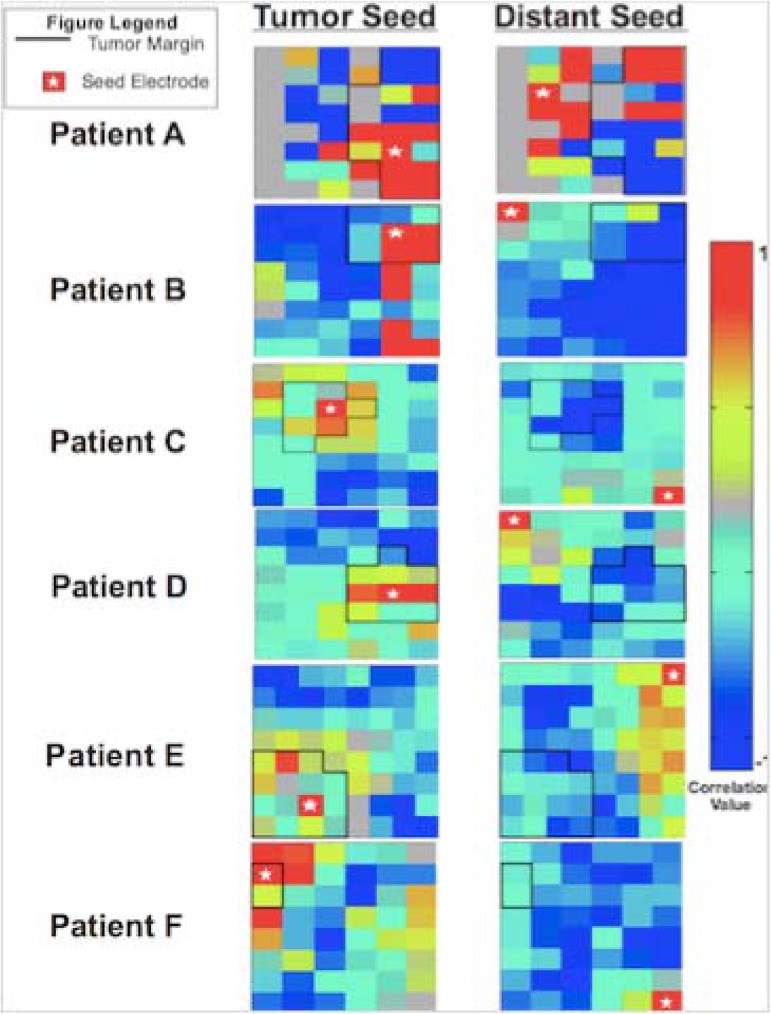
Patient specific correlation matrices demonstrating representative correlation maps with seeds placed centrally within each patient’s tumor (left column) and remotely within distant cortex (right column) for each individual subject.

Despite maintenance of connectivity overall, there is a perturbation in the cortical surface topography of correlation values relative to neoplastic tissue compared to non-neoplastic tissue. Said another way, the topographic structure of tumor and distant cortical sites are similar in distribution but separate in morphology. Spatial correlation comparisons between surface topographies for both tumor and distant seed electrodes demonstrate an internally consistent, positively correlated, topographic structure within the tumor compared to its surroundings that is absent when selecting for a distant electrode (Fig 5). Five of the six patients were found to have statistically significant reciprocal topographies between tumor and distant seed maps by spatial correlation analysis. This suggests that areas infiltrated by tumor show correlated activity that is separable from surrounding cortical activity. For alpha = 0.05 / 6 = 0.00833 (Bonferroni corrected for N = 6):

Patients A, B and C significant at p < 0.001

Patients D and E significant at p < 0.005

Patient F did not show any statistically significant relationship between tumor and distant seed maps.

## Discussion

Using invasively acquired electrophysiologic signal from human subjects, this study characterizes the impact of a high-grade primary glial neoplasm on cortical physiologic phenomena. The physiologic behavior of electrodes overlying neoplastic tissue was compared to that of electrodes overlying distant, normal tissue. Tumor infiltrated tissue appeared to have a reduced global power in the majority of patients, while maintaining an infra-slow scale network structure. Interestingly, this maintained network structure appeared predominantly limited to the region involved with the tumor. Taken together, these findings support the possibility of a “de-learning” phenomenon in which poorly functioning tissue alters its connectivity patterns to which it is ascribed prior to development of the neoplastic process and this becomes progressively sequestered.

### Physiologic implications

The preservation of connectivity within the tumor as demonstrated by an unchanged mean correlation value between electrodes related to the underlying neoplasm and those related to distant cortical tissue indicates that the capability for connectivity is maintained at a cellular level despite the aggressive and infiltrative behavior of the underlying high grade glial neoplastic process. The negative-shift of these SCPs likely reflects the result of slow rhythmic depolarization of apical dendrites in superficial cortical layers [32, 33]. Thus, despite the tumor infiltrating more deeply in the white matter and even transgressing the gray matter the more superficial mechanisms of synaptic homeostasis seem to be preserved. This is in keeping with findings in peritumoral regions in a murine glioma model in which neurons are found to demonstrate functional changes including increased spontaneous activity which was reversible with administration of cytotoxic necrotizing factor 1 suggesting reversibility to these changes in the peritumoral area compared to the tumor core[34]. That said, on the topographic level it appears that there is an alteration in network structure that appears to be constrained to the region of the tumor as defined by covariance analysis of the ECoG signal. Namely, that the resting state network of internal intratumoral connectivity, is limited to the general area of the tumor itself.

On the spectral scale, there also are perturbations. There is the global power reduction within the tumor when compared to its surroundings. This reduction in overall power across frequency bands suggests that both thalamo-cortical and cortico-cortical processing is limited within the neoplastic process when compared to its surroundings [35–37]. In addition to this global power reduction there is also a subtle flattening of the μβ peak in patients D & F. Mu (8-12Hz) and beta (18-26Hz) rhythms are oscillations that are thought to represent post-synaptic potentials associated with thalamocortical modulation of cortex [38]. These rhythms tend to be spectrally confined and anatomically broad. Their absence in power in electrodes overlying tumor-infiltrated regions suggests that impairment in the thalamic interactions with more superficial cortical regions has occurred. Given that gliomas are often found primarily in the white matter and likely to impact cortical connections with deeper structures, the loss in narrow band power peaks also provides “lesional” support for these mu and beta rhythms reflecting a disconnection between the thalamus and cortex. In the absence of the narrow band spectral oscillator peaks the residual signal is a lower power broad band signal that follows a power law distribution. This likely reflects residual asynchronous neuronal firing in cortex [28].

Taken together, the networked topography and the spectral analysis show the region of brain invaded by the tumor to be relatively homogeneous. While it is important to note that the physiologic measurement was taken at a single point in time (immediately prior to tumor resection), that the infiltrative nature of the glial neoplastic process may lead to progressive regional functional decline which manifests as intratumoral physiologic isolation is intriguing to consider. This is in keeping with the graded changes seen in resting state fMRI data in the presence of a glioblastoma where the tumor and contralateral internal control timeseries were significantly different from each other with the timeseries of the non-enhancing peritumoral tissue more closely resembling that of the tumor than that of the contralateral internal control[20]. This physiologic sequestration notion supports a de-learning phenomenon occurring within the tumor. Previous reports indicate that a degree of plasticity exists within SCP network connectivity [14, 39]. The corollary of that phenomenon appears to be functioning here in which the tumor creates a de-learning process. In the same way that repetitive task exposure and execution can strengthen SCP connectivity between two regions, the neoplastic invasion appears to reduce overall activity (e.g. global power reduction), reduce thalamocortical interaction (loss of mu-beta peaks), and reduce SCP connectivity between the tumor and distant regions. Cumulatively, this leads to maintained intratumoral connectivity and functional sequestration.

### Clinical implications

Approximately 20,000 new primary glial neoplasms are diagnosed each year [40]. Of these, roughly half are aggressive, grade IV glioblastomas [41]. Overwhelmingly, the most common locations for these tumors to occur are within the frontal, temporal and parietal lobes [42]. These cortical regions largely comprise functional cortex involved in both motor and language systems, among others. Many of these tumors are in close proximity to these functional cortical regions. This situation often requires employing mapping techniques during their resection in order to maintain safe distance from adjacent functional cortex. Burgeoning techniques for mapping functional areas are currently emerging that use endogenous electrophysiology either during a task or at rest to complement more classic methodologies of electro-cortical stimulation (ECS)[43–46]. All the early demonstrations of these techniques applied during the resting state, however, have used invasively monitored epilepsy subjects without distorting brain lesions.

For task-based applications, amplitude modulation in higher gamma frequency bands has been used to identify focal areas associated with cortical activation. Activity in the high gamma frequency range (> 40 Hz) are thought to be produced by local cortical circuits [47]. Because of the anatomically constrained nature of high frequency amplitude modulation, these gamma rhythms have been focused on as the optimal signal for use in task-based clinical brain mapping [48, 49]. In the context of a brain tumor and the demonstration of reduced power across all frequencies it will be important to test if the sensitivity and specificity determined in non-lesional human cortex will still be maintained. Task independent mapping techniques have also been employed [50]. As mentioned, the cortical physiology that most correlates with fMRI-defined resting state networks are covariant regions defined by very low frequency oscillations (< 0.5Hz), known as slow cortical potentials. [51] Given that these networks are durable through sleep wake cycles, and anesthesia [52–54], they were evaluated as a possible tool for localizing eloquent cortex. Breshears et al., demonstrated that these SCP defined networks identified sensorimotor cortex using a data-driven approach in patients under anesthesia and awake with a high sensitivity, suggesting that resting-state networks may be useful for tailoring subsequent stimulation mapping. This must be guided however by the knowledge that BOLD activations are reduced in the setting of an infiltrating glioma [55, 56]. If the neoplastic tissue is anatomically sequestered from surrounding, non-neoplastic cortex, it remains an open question as to whether it would be safe to resect.

### Limitations

The limitations of this study include a relatively small sample size of six patients. A second limitation was limited electrode grid coverage. Each patient’s grid coverage was dictated exclusively by clinical circumstances alone, therefore our findings should only be interpreted in the context of limited electrode grid coverage. Finally, our findings are applicable exclusively to high-grade glial neoplasms. As such, the consistency of cortical physiologic phenomena in lower grade glial neoplasms and metastatic disease still remains unknown.

## Conclusions

We report a unique assessment of invasive human cortical physiology related to glioblastoma. Observations from our analysis include the maintenance of the capability for connectivity within the tumor but a physiologic sequestration of the tumor from the surrounding cortex coupled with an overall reduction in global power of these regions. These findings suggest a possible de-learning phenomenon that may result in a functional isolation of the region of brain invaded by the tumor. In addition to providing insight into how the brain accommodates a region of “defunctionalized” cortex, these findings may have important clinical implications including applications for emerging techniques in brain mapping using endogenous cortical physiology.

## Supporting information

S1 File PatientAdata.mat. Patient A data file.
(MAT)

S2 File PatientAparams.mat. Patient A acquisition parameters.
(MAT)

S3 File PatientBdata.mat. Patient B data file.
(MAT)

S4 File PatientBparams.mat. Patient B acquisition parameters.
(MAT)

S5 File PatientCdata.mat. Patient C data file.
(MAT)

S6 File PatientCparams.mat. Patient C acquisition parameters.
(MAT)

S7 File PatientDdata.mat. Patient D data file.
(MAT)

S8 File PatientDparams.mat. Patient D acquisition parameters.
(MAT)

S9 File PatientEdata.mat. Patient E data file.
(MAT)

S10 File PatientEparams.mat. Patient E acquisition parameters.
(MAT)

S11 File PatientFdata.mat. Patient F data file.
(MAT)

S12 File PatientFparams.mat. Patient F acquisition parameters.
(MAT)

## Abbreviations

ECoG: electrocorticography
MRI: magnetic resonance imaging
SCP: slow cortical potential
Hz: hertz
mm: millimeters
BOLD: blood oxygen level dependent
ROI: region of interest
